# Development of machine learning models for predicting properties of carbon materials and design of process conditions for production of materials with desired multiple properties

**DOI:** 10.1007/s44211-026-00890-5

**Published:** 2026-03-11

**Authors:** Masayoshi Matsubara, Ryo Sasaki, Jun P. Takahara, Shinji Moritake, Yasuyuki Harada, Hiromasa Kaneko

**Affiliations:** 1 Department of Applied Chemistry, School of Science and Technology, Meiji University, 1-1-1 Higashi-Mita, Tama-ku, Kawasaki, Kanagawa 214-8571 Japan; 2 Mitsubishi Chemical Corporation, 1-1 Marunouchi 1-chome, Chiyoda-ku, Tokyo, 100-8251 Japan

**Keywords:** Needle coke, Pitch coke, Machine learning, Process design, Multi-objective optimization

## Abstract

Cokes are an essential material in the iron and steel industry, and widely used as a fuel and for reducing iron ore. The product properties of cokes are influenced by raw materials and process conditions, which in turn affect its performance. Because raw material components vary depending on the origin and production period, product properties can fluctuate even under the same process conditions, and thus, it is necessary to design appropriate process conditions for each raw material. The objective in this study is to construct machine learning models that predict product properties from raw materials and process conditions using data sets measured in needle and pitch coke production processes, and to design appropriate process conditions that achieve desired values of properties by inverse analysis of the constructed models. To select appropriate process conditions and their time delays for a data set, we propose a method called NSGA-II-VDS, combining elitist non-dominated sorting genetic algorithm (NSGA-II) and genetic algorithm-based process variable and dynamics selection (GAVDS), for multiple product properties, and confirmed that the proposed NSGA-II-VDS could construct models with higher prediction accuracy than the conventional GAVDS. Furthermore, we constructed models using a data set where time-series data was interpolated based on the maximum batch time for each process condition for a batch process data set. By inputting time-series data generated virtually into the constructed models for raw material information for a target product, we successfully designed process conditions predicted to achieve the desired product property values by selecting time-series data with promising predicted product quality values.

**Graphical abstract:**

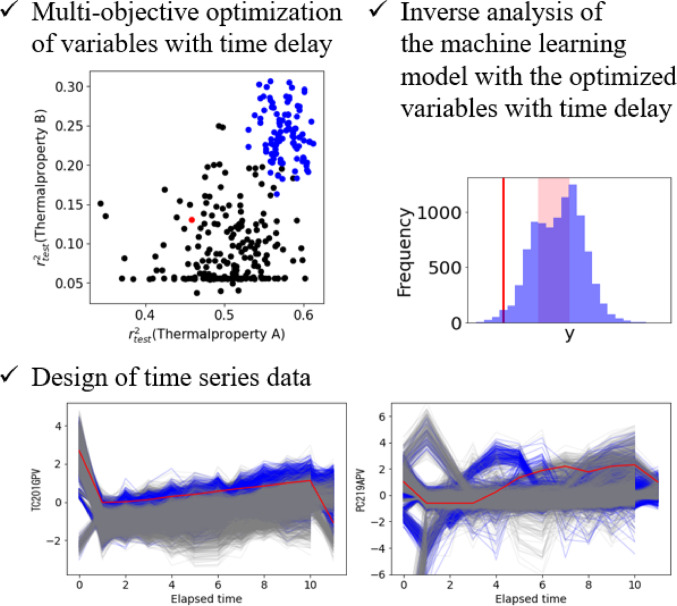

## Introduction

Cokes are an essential material mainly in the steelmaking industry and is widely used as a fuel and reducing material to reduce iron ore [1, 2]. Their basic properties of high carbon content, excellent strength, and low impurity content directly affect the efficiency and quality of the entire steelmaking process [3–6], and thus, appropriate quality control of cokes is required [7]. The quality of cokes is greatly influenced by the raw materials and process conditions during the manufacturing process [8–10]. The raw material composition is represented by a mixture of several raw material components, and the raw material composition varies depending on its origin and year [11, 12].

When cokes are produced under certain process conditions, different raw material components can cause the product to be out of specification and the quality to be inconsistent [13–16]. To maintain coke quality within the target range, process conditions must be varied appropriately for each raw material. However, designing appropriate process conditions is difficult because there are a vast number of parameters in most manufacturing processes, and it is not enough to focus on just one of them; they are affected with each other, and should be considered simultaneously. Currently, process conditions are designed based on experience, operation, and evaluation of product properties. Based on this experience, process conditions are designed again and again, aiming to design appropriate process conditions through trial-and-error, which incurs the cost of repeated operations.

In material production processes, machine learning models have been constructed to predict product properties from process parameters [17–19]. Product properties of cokes, which are the variables to be finally measured, are objective variables Y, and raw material properties and process conditions such as temperature and pressure are explanatory variables X, and then, machine learning model Y = f(X) is constructed to represent the relationship between X and Y [20]. By inputting virtually generated candidates of X into the constructed model, Y values are predicted, which makes it possible to predict product properties in advance without conducting experiments and operations on all candidates.

The objective of this study is to develop predictive machine learning models predicting multiple product properties using two data sets of different coke types: needle and pitch cokes, and to design process conditions that will result in the desired values of multiple product properties. The first data set (Data set 1) is provided from a needle coke production process in which there are multiple steps in the process, and X is recorded at each process time, but X at all measurement times does not necessarily affect Y. Therefore, the genetic algorithm-based process variable and dynamics selection (GAVDS) [21], a method for regionally selecting combinations of X and their measurement times that affect Y using a genetic algorithm, is applied to select appropriate process variables and their time delays. Because there exist two y-variables in Data set 1, we propose a method that combines the multi-objective optimization method, the elitist non-dominated sorting genetic algorithm (NSGA-II) [22] and GAVDS, called NSGA-II-based process variable and dynamics selection (NSGA-II-VDS). This proposed method develops a model with high prediction accuracy for multiple product properties using combinations of X that affect Y and their measurement times.

The second data set (Data set 2) is provided from a pitch coke production process, which is a batch process, and a product property is measured at the end of each batch. The products are often manufactured at different batch times to stabilize product quality [23]. Therefore, we employ the method of interpolating values based on the largest batch time for each manufacturing condition and the method of converting time series data for each batch [24, 25] to construct a model using X that considers different batch times. In addition, because there exist missing values in Data set 2, missing values are complemented using iterative Gaussian mixture regression (iGMR) [26] and compared with the method of removing process conditions with missing values.

Furthermore, by performing the inverse analysis of the constructed models, it is possible to propose process conditions that will yield the desired property values for a certain raw material. T-Gen [27] was employed to generate virtual time series data for candidates of X in the inverse analysis. Cross-validated permutation feature importance (CVPFI) [28] was used to calculate the importance of X of the model and to identify changes over time in process conditions that are important for the prediction of Y. To validate the inverse analysis in this study, we run the inverse analysis on a sample of raw materials manufactured in the past and compare the result to the actual measured value of Y. By performing the proposed inverse analysis, process conditions that exhibit more appropriate Y values than the actually measured Y values could be designed.

## Methods

### Data sets

**Fig. 1:**
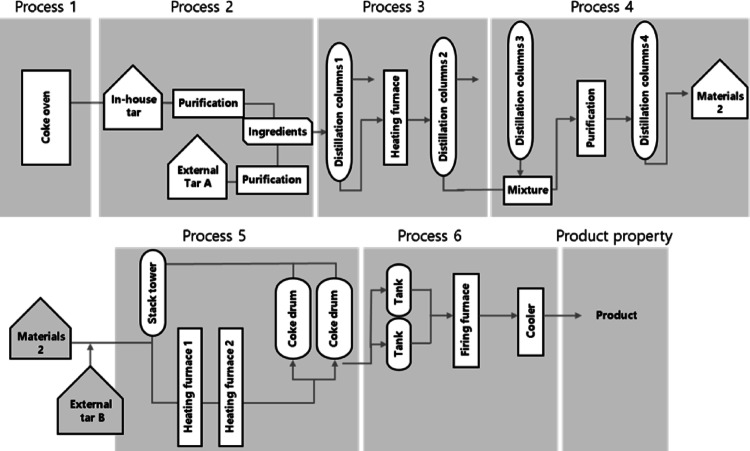
Needle coke production process

Two coke production data sets were collected in Mitsubishi Chemical Corporation. Data set 1 is a data set recorded in needle coke production from 2012 to 2023. The basic concept of the needle coke production process is shown in Fig. 1. In Process 1, coal is produced as raw material and the production parameters are adjusted to produce coke for steelmaking as the main product. As by-products, tar and gas are obtained in this process. Needle coke is produced from this byproduct, tar, as the starting material. Needle coke produced is made from coal tar from coke ovens [29]. Process 2 is the process of removing impurities by mixing with another tar. Process 3 is the process of removing the tar and removing the light content to obtain the raw pitch for the carbon material. Process 4 is the process of reforming the raw material pitch to produce raw materials for each brand. Process 5 is the process of coking, which refers to heating the raw material to turn the liquid soft pitch into a solid. In addition, external tar is mixed into the raw material in Process 5. Process 6 is the process of calsign, which is the process of turning the solidified raw material through coking into a carbon product by heating it at a higher temperature. The parameters of each process can be divided into two categories: process conditions and material parameters, which are collectively referred to as X. Operating parameters are 415 variables such as temperature, flow rate, pressure, and gas volume, while material parameters are 157 variables related to molecular weight, molecular structure, specific gravity, and impurities. In this study, the variable-and-dynamics selection (time-window / time-delay selection) is applied to the time-indexed operating parameters that are recorded as a time series. In contrast, material parameters are treated as static descriptors for each sample and are not subjected to time-window selection. This clarification is provided to explicitly indicate which explanatory variables are optimized by GAVDS/NSGA-II-VDS. The product quality, Y, is represented by two thermal properties. There are 535 samples for which Y was measured.

The raw material is manufactured in each step of the process, starting with Process (1) Data are obtained from each process, and are recorded with the date. Because the plant is large, the time from the raw material to the end of the entire process is about one month. Considering the raw material at a certain point in the continuous process, for example, the date when it was produced in Process 1 is different from the date when it was produced in Process (2) In Processes 5 and 6, the process is conducted at the same date. In Process 5, intermediate materials manufactured in Processes 1 through 4 are stored in a tank. In Process 6, intermediates manufactured in Process 5 are stored together. Process 5 and Process 6, and Process 6 and Y are linked by lot numbers.

**Fig. 2:**
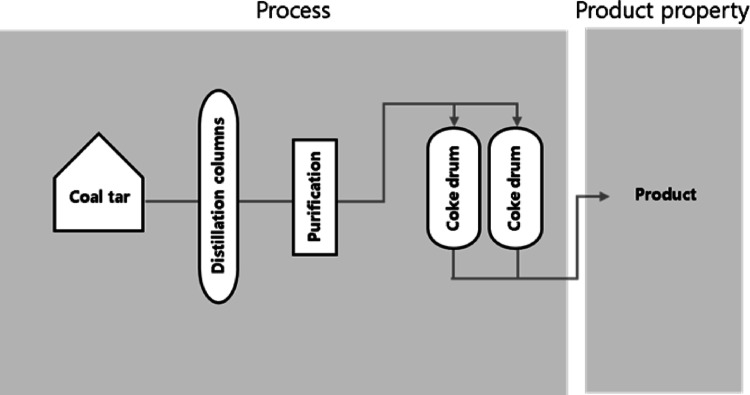
Pitch coke production process

Data set 2 is a data set recorded in a pitch coke production process from 2007 to 2023. The plant is operated in a batch process and a product property is measured for each batch. The process flow is shown in Fig. 2. First, the raw coal tar is distilled to separate the light components. Next, refining is performed to separate the components that inhibit crystallization. Finally, coking is performed to coke the pitch at high temperature. The parameters can be divided into two categories: process conditions and raw material properties. The process conditions are 20 controllable variables such as temperature, flow rate, pressure, and flow rate, and the raw material properties are six variables related to compounds and impurities. For Data set 2, the time-series characterization (interpolation/DTW/DFT) is applied to the process-condition variables along the batch elapsed time, whereas the raw-material properties are treated as batch-level static variables and are not subjected to time-window selection. The product property, Y, is volatile matter. The number of samples for which Y was measured is 47.

Pitch coke production process data exist as a tensor data set of (samples) × (process conditions) × (elapsed time). It is the time from the start point of manufacturing to the end point of the batch process. In this study, the time axis was expanded for each process condition to prepare a two-dimensional data set of (samples) × (process conditions and elapsed time). Because the time from the manufacturing start point to the end point of the batch process and the batch time length are different, there exist no data for samples with a batch time shorter than the maximum batch time.

### Regression model

The regression analysis method used in this study is given as follows:

- Ordinary least squares regression (OLS) [30].
- Partial least squares regression (PLS) [31].
- Ridge regression (RR) [32].
- Least absolute shrinkage and selection operator (LASSO) [33].
- Support vector regression with a Gaussian kernel (NLSVR) [34].
- Decision tree (DT) [35].
- Random forests (RF) [36].
- Gradient boosting decision tree (GBDT) [37].
- Extreme gradient boosting (XGB) [38].
- Light gradient boosting machine (LGBM) [39].
- Gaussian process regression (GPR) [39].
- Gaussian mixture regression (GMR) [40].
- Variational Bayesian Gaussian mixture regression (VBGMR) [41].

The regression analysis method with the highest prediction accuracy when train-test split or double-cross-validation (DCV) was performed on each data set was used as the regression analysis model for that data set.

### GAVDS

In GAVDS/NSGA-II-VDS, candidate solutions are represented on a variable–time grid, where the time-delay axis is discretized according to the data-recording interval (e.g., day-based lags for date-recorded variables). “Selection area” refers to a contiguous time window on this grid. The parameter “maximum selection area width” specifies the maximum width of a selected time window (in lag steps), and “number of areas” specifies how many such windows are selected in one solution. These parameters control the sparsity and locality of the selected variable–time regions.

To construct a model for predicting product properties of Data set 1, GAVDS, a method for selecting process conditions and their time delays that affect Y, was used. First, a data set is prepared with data on process conditions for each time delay to be considered from the point in time when the product properties were measured. The maximum time delay *t*_*i*_ from the point in time when the product properties were measured for the process is determined in advance, and the measured data from the date when the product properties were measured up to *t*_*i*_ ago are combined as new variables. Thus, if the number of process conditions is *m* and the maximum time delay to be considered is *t*, the number of X to be prepared for GAVDS is *m*×*t*. Regression analysis is performed between the variables selected from the prepared data set and Y to obtain individuals with high coefficients of determination r^2^ for a validation data set. The GAVDS calculation is performed repeatedly because the local optimum solution can be provided in GA. DEAP [42] was used to calculate the GA.

### NSGA-II

NSGA-II is one of the optimization algorithms and is an effective method for searching for Pareto optimal solutions when more than one fitness function exists. Pareto optimal solutions are a group of solutions that are not superior to or inferior to other solutions. In this study, the coefficients of determination r^2^ of the two objective variables after cross-validation in Data set 1 are used for the fitness function, and thus, the solution with the higher r^2^ of the two Y variables is selected. The NSGA-II algorithm is given as follows:

1. Randomly generate an initial population.
2. Evaluate fitness functions.
3. Rank individuals by non-dominated sorting.
4. Select parent population by tournament selection.
5. Apply crossover and mutation.
6. Merge parent and child populations.
7. Perform nondominated sorting.
8. Select next generation individuals using rank and crowding distance.
9. Repeat 2–8 up to a pre-determined number of times.

DEAP [42] was used to calculate the NSGA-II.

### Time series data with different lengths for each batch

The following three interpolation methods are used to prepare a data set based on the longest batch of time-series data, as there exist parts of the data set that have no values.

- Interpolation with zero (Zero interpolation).
- Interpolation with the average value of each batch process (Average interpolation).
- Interpolation with the final value (end point) of each batch process (Final value interpolation).

When the number of process conditions is *s* and the length of the longest batch *is t*, the number of X is *s* × *t*.

In addition to the interpolation method, we employ dynamic time warping (DTW) [43], which calculates similarity between time series data of different lengths, and discrete Fourier transform (DFT) [44], which transforms time series data into frequency domain features. When the number of process conditions is *s* and the number of samples is *n*, the number of X is *s* × *n* in DTW. When DFT is used, a DFT transformation is performed for each manufacturing condition for each sample, and the real and imaginary parts of the complex number representation are used as feature values. When the number of process conditions is *s* and the number of complex numbers is *c*, the number of X is *s*× *c* × 2 in DFT. Because there exist samples with batch data sizes of 11 and 12, the number of frequencies *N* to be transformed by DFT was considered to be 11 and 12.

### Inverse analysis of time series data of process conditions

By fixing raw materials at any values and setting X, and performing the inverse analysis of the model, it is possible to design process conditions that will yield the desired product property values when raw materials are provided. First, regression analysis is performed using a data set to construct the optimal regression model for predicting Y from X. Next, the raw material is fixed as a certain sample, and 10,000 samples of time-series data of process conditions generated by T-Gen are used to predict the product property values using the model that has already been constructed. By selecting the sample with the best predicted Y value from the 10,000 samples, the optimal process conditions can be designed.

## Results and discussion

### Needle coke production process

Using Data set 1 of the actual needle coke manufacturing process, machine learning models were developed to predict product properties from dynamic process data. 400 samples were used as training data and 135 samples as test data. To optimize process conditions and their time delays, the maximum time delay considered was 30 days for Processes 2–4 and the last 4 lots for external raw materials. r^2^, which represents the predicted performance of one product property predicted with GAVDS, was used as the evaluation function to optimize the time delay and its process conditions. r^2^ representing the predicted performance for two product properties when X is selected by NSGA-II-VDS and the selected X is used was used as the evaluation function to optimize the time delay and its process conditions Tables 1 and 2.

**Table 1:** GAVDS parameter settings in the needle coke production process

Setting	Parameters
Number of generations	500
Number of populations	100
Maximum time delay (day-based lags)	Processes 2–4: 30 (days) External tar B: 4 (days)
Maximum selection area width	10 (days)
Number of areas to be selected	10–100 in increments of 10
Probability of crossover	50%
Function of crossover	Two point crossover
Probability of mutation	20%
Function of mutation	Flip the value of the attributes of the input individual and return the mutant
Evaluation function	r^2^ after 5-fold cross validation
Regression method	Support vector regression with a Gaussian kernel

**Table 2:** NSGA-II-VDS parameter settings in the needle coke production process

Setting	Parameters
Number of generations	250
Number of populations	100
Maximum time delay (day-based lags)	Processes 2–4: 30 (days) External tar B: 4 (days)
Maximum selection area width	10 (days)
Probability of crossover	90%
Function of crossover	Simulated binary crossover
Probability of mutation	100%
Function of mutation	Polynomial bounded mutation
Evaluation function	r^2^ after 5-fold cross validation
Regression method	Support vector regression with a Gaussian kernel

**Fig. 3:**
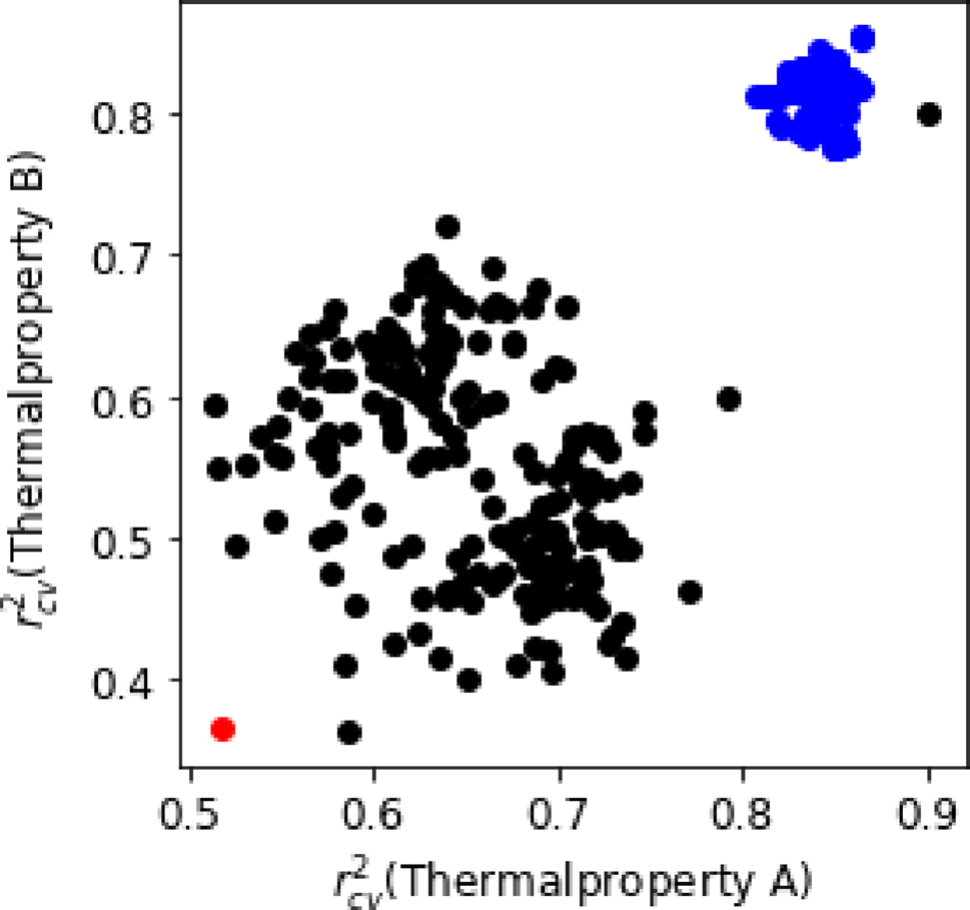
r^2^ after cross-validation for thermal properties A and B. The red, black, and blue points indicate the results of all the X variables, GAVDS, and NSGA-II-VDS in the needle coke production process

NSGA-II-VDS provided multiple Pareto solutions and 110 optimal solutions were obtained in a single calculation. One optimal solution is a combination of process conditions and their time delays, and 110 optimal solutions represent 110 patterns of process conditions and their time delay combinations. The results of r^2^ after cross-validation are shown in Fig. 2 as the results in original X before the selection of process conditions and their time delays, the 100 results in X after being selected with GAVDS, and the 110 results in X after being selected with NSGA-II-VDS. The GAVDS models of the black points had higher r^2^ values for both Y variables than the all-x model of the red point. Furthermore, the proposed NSGA-II-VDS models of the blue points had higher r^2^ for both Y variables than the GAVDS models. Although the conventional GAVDS model had a solution equivalent to NSGA-II-VDS as shown at the upper right point in Fig. 3, the NSGA-II-VDS models were more stable than the GAVDS models in obtaining solutions with higher r^2^ values for the two objective variables.

**Fig. 4:**
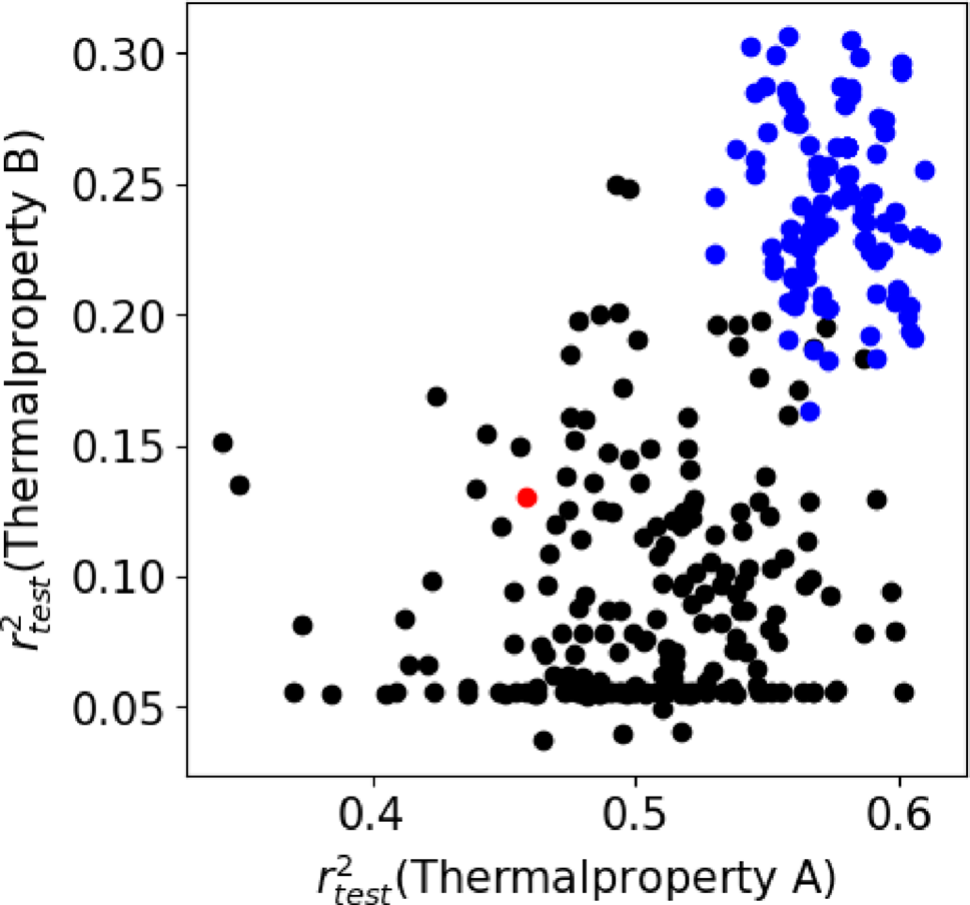
r^2^ of the test data for thermal properties A and B. The red, black, and blue points indicate the results of all the X variables, GAVDS, and NSGA-II-VDS in the needle coke production process

Regression models were constructed using 100 results of X selected with GAVDS and 110 results of X selected with NSGA-II-VDS to predict the test data of the 135 samples. The prediction results are shown in Fig. 4. Comparing the prediction results of the test data with the all-X model of the red point and those with the GAVDS models of the black points, several solutions with higher prediction accuracy were obtained by the GAVDS models than by the all-variable model. Comparing the prediction results of the test data with the GAVDS models of the black points and those with the NSGA-II-VDS models of the blue points, the NSGA-II-VDS models showed higher prediction accuracy for most solutions. For the optimal solution, the GAVDS model had lower prediction accuracy for the test data, because there exist no black points in the upper right corner of Fig. 4, suggesting that overfitting occurred due to GAVDS. On the other hand, the prediction results for the test data by the proposed NSGA-II-VDS model were as stable as the optimization results and resulted in high r^2^ of the test data for the two Y variables. It was confirmed that the proposed NSGA-II-VDS can select X to construct models with high prediction accuracy for multiple Y variables.

**Fig. 5:**
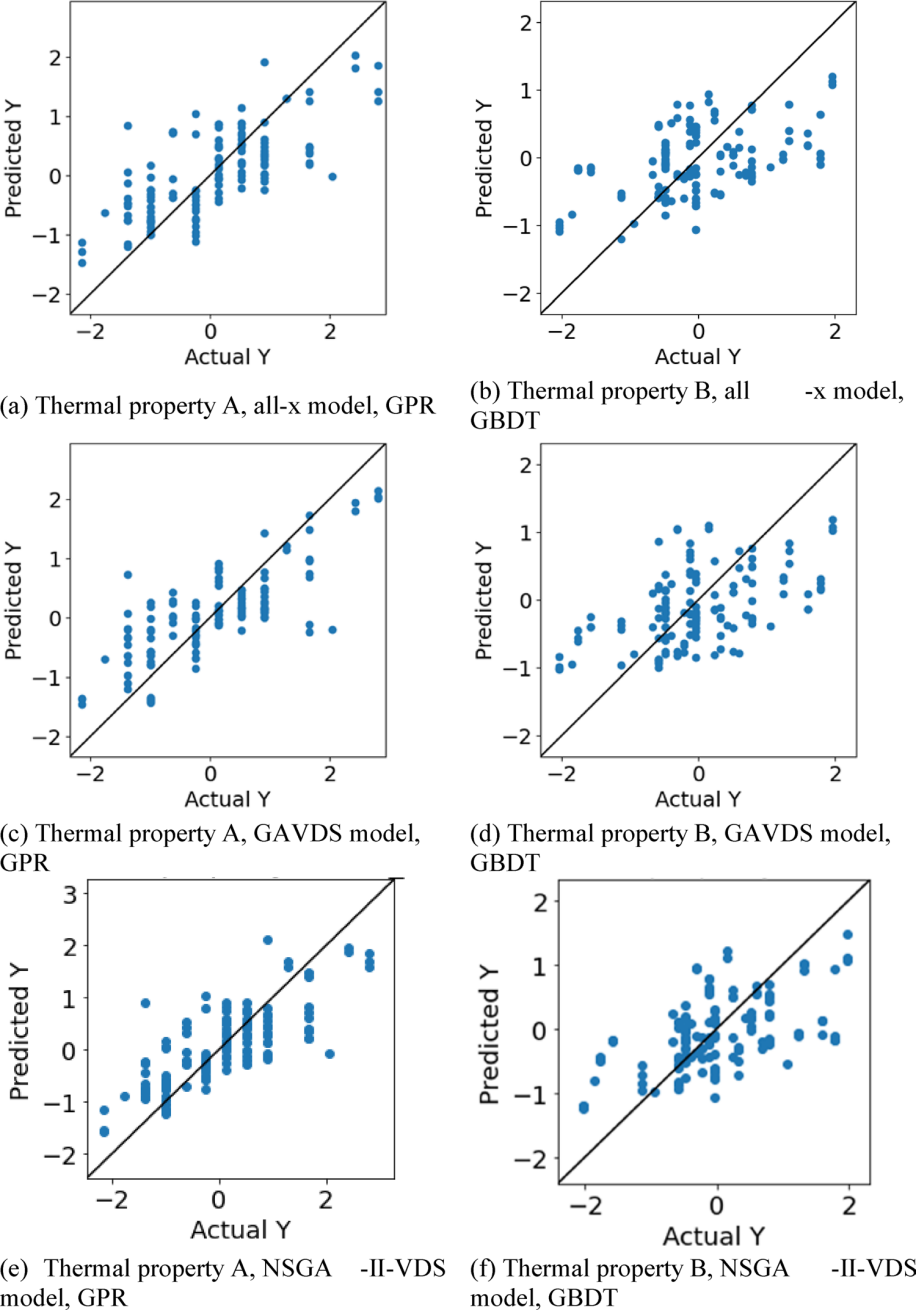
Scatter plots of the measured Y values and the predicted Y values for the test data in the needle coke production process. (**a**) Thermal property A, all-x model, GPR, (**b**) Thermal property B, all-x model, GBDT, (**c**) Thermal property A, GAVDS model, GPR, (**d**) Thermal property B, GAVDS model, GBDT, (**e**) Thermal property A, NSGA-II-VDS model, GPR, (**f**) Thermal property B, NSGA-II-VDS model, GBDT

Figure 5 shows the plots of the measured Y values and the predicted Y values for the test data by the all-variable model, GAVDS model, and NSGA-II-VDS model, and Table 3 shows the evaluation indexes for the test data. For thermal property A, the GAVDS and NSGA-II-VDS models showed higher accuracy than the all-x model. On the other hand, for thermal property B, although the GAVDS models showed no improvement in prediction accuracy, which would be because the process conditions selected by GAVDS and its time delay resulted in the optimization for the prediction of thermal property A, the prediction accuracy was improved by using the NSGA-II-VDS models compared with that of the all-x model. From Fig. 5(a)(e) for thermal property A, the samples with higher and lower measured values in the NSGA-II-VDS model than in the all-x model were closer to the diagonal line. This indicated that the NSGA-II-VDS models achieved high prediction accuracy. For thermal property B, Fig. 5(b)(f) showed that the samples with higher and lower measured values in the NSGA-II-VDS models were closer to the diagonal, indicating that the model had high prediction accuracy.

**Table 3:** Evaluation indexes for the test data for thermal properties A and B for the all-x model, GAVDS model, and NSGA-II-VDS model in the needle coke production process

y	Model	Method	*r* ^2^
Thermal property A	All-x	GPR	0.527
	GAVDS	GPR	0.602
	NSGA-Ⅱ-VDS	GPR	0.601
Thermal property B	All-x	GBDT	0.260
	GAVDS	GBDT	0.248
	NSGA-Ⅱ-VDS	GBDT	0.293

### Pitch coke production process

Using the actual batch manufacturing process data set for a specific brand of pitch cokes (Data set 2), machine learning models were developed to predict product properties from raw materials and process conditions, and to design process conditions that are estimated to achieve the target product properties. First, a data set was prepared using 47 hourly data samples of actual batch manufacturing process data. The time-series data was characterized using five methods: zero interpolation, mean value interpolation, final value interpolation, DTW, and DFT. The data set contained X variables with missing values, one variable for raw material properties and one variable for controllable process conditions, and was created in two ways: by deleting the variables and by supplementing with iGMR. Using the created data set, regression analysis with double cross-validation (DCV) was performed for each time series data characterization method. In DCV, the number of outer folds was the number of samples and that of inner folds was five. The evaluation index for the prediction accuracy of each method was r^2^ by DCV.

**Fig. 6:**
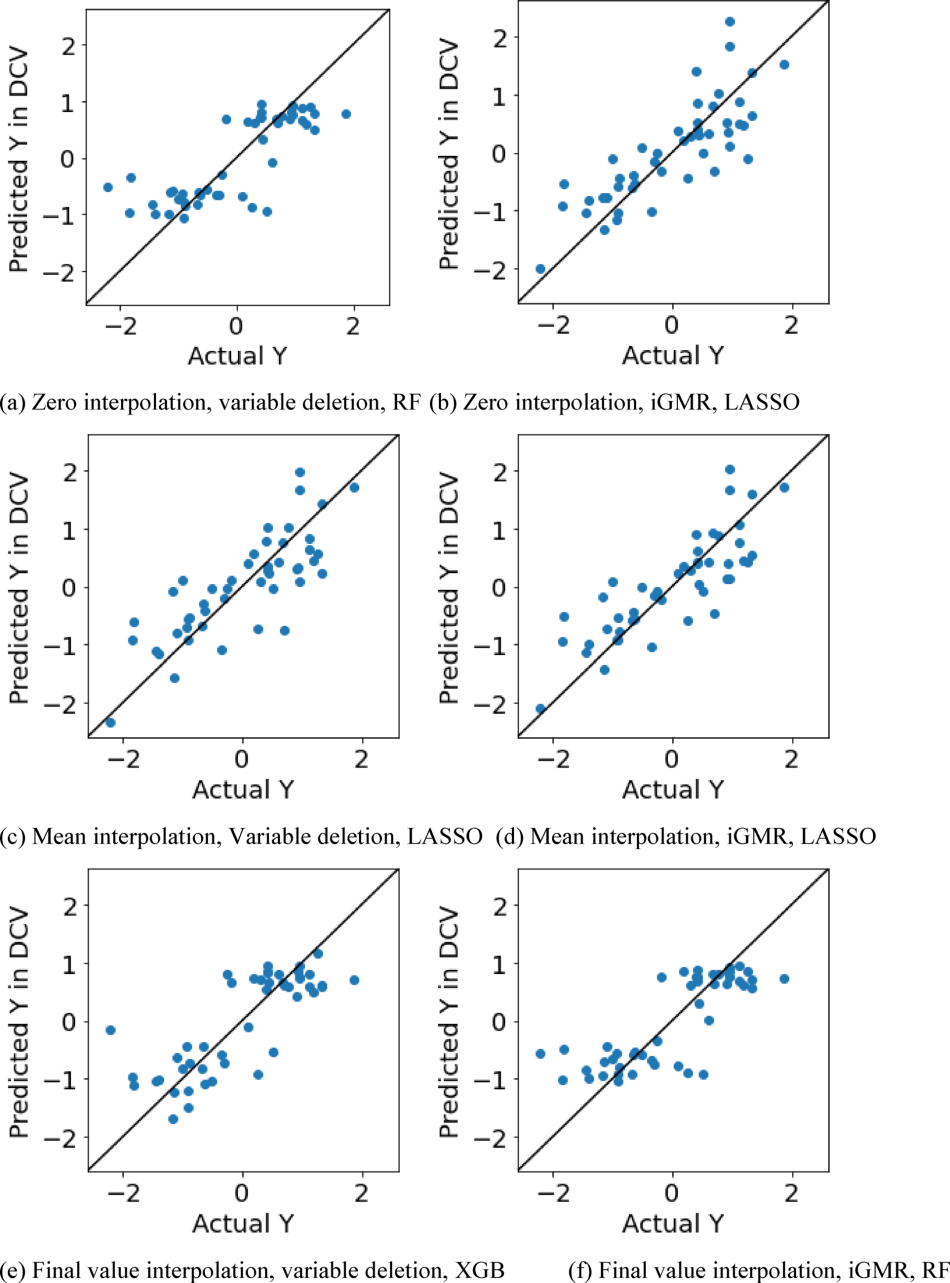

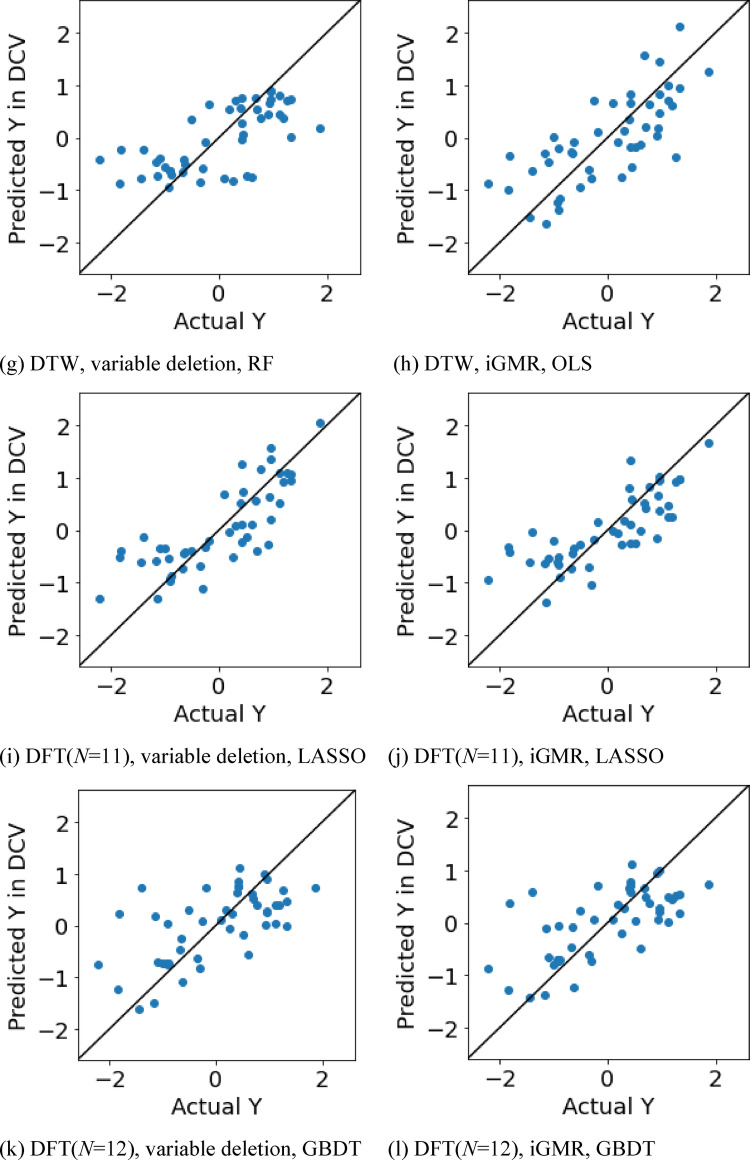
Scatter plots of measured Y values vs. predicted Y values after DCV in the pitch coke production process. (**a**) Zero interpolation, variable deletion, RF, (**b**) Zero interpolation, iGMR, LASSO, (**c**) Mean interpolation, Variable deletion, LASSO, (**d**) Mean interpolation, iGMR, LASSO, (**e**) Final value interpolation, variable deletion, XGB, (**f**) Final value interpolation, iGMR, RF, (**g**) DTW, variable deletion, RF, (**h**) DTW, iGMR, OLS, (**i**) DFT(*N* = 11), variable deletion, LASSO, (**j**) DFT(*N* = 11), iGMR, LASSO, (**k**) DFT(*N* = 12), variable deletion, GBDT, (**l**) DFT(*N* = 12), iGMR, GBDT

Table 4 shows the evaluation indexes of prediction accuracy after DCV for the methods with the highest r^2^ after DCV among the regression analysis methods shown in 2.2, and Fig. 6 shows the plots of measured Y values and predicted Y values. The model with the highest r^2^ in Table 4 corresponded to mean interpolation as the time series data characterization method, iGMR to handle missing values, and LASSO as the regression method. Overall, the data set with missing values complemented by iGMR showed higher prediction accuracy; for DFT, the prediction accuracy varied greatly depending on the value of *N*. To improve prediction accuracy of the models, it is necessary to set the value of *N* appropriately to obtain the features important for predicting Y values. From Fig. 6(d) for the model with the largest r^2^, the samples were close to the diagonal line for high and low measured values, indicating that the prediction was highly accurate.

**Table 4:** Evaluation indexes of prediction performance of regression models in DCV in the pitch coke production process

Time series data characterization method	Handling with missing values	Regression method	*r* ^2^
Zero interpolation	Variable deletion	RF	0.635
	iGMR	LASSO	0.655
Mean interpolation	Variable deletion	LASSO	0.640
	iGMR	LASSO	0.682
Final value interpolation	Variable deletion	XGB	0.629
	iGMR	RF	0.638
DTW	Variable deletion	RF	0.451
	iGMR	OLS	0.522
DFT(*N* = 11)	Variable Deletion	LASSO	0.631
	iGMR	LASSO	0.609
DFT(*N* = 12)	Variable deleted	GBDT	0.386
	GMR	GBDT	0.434

**Fig. 7:**
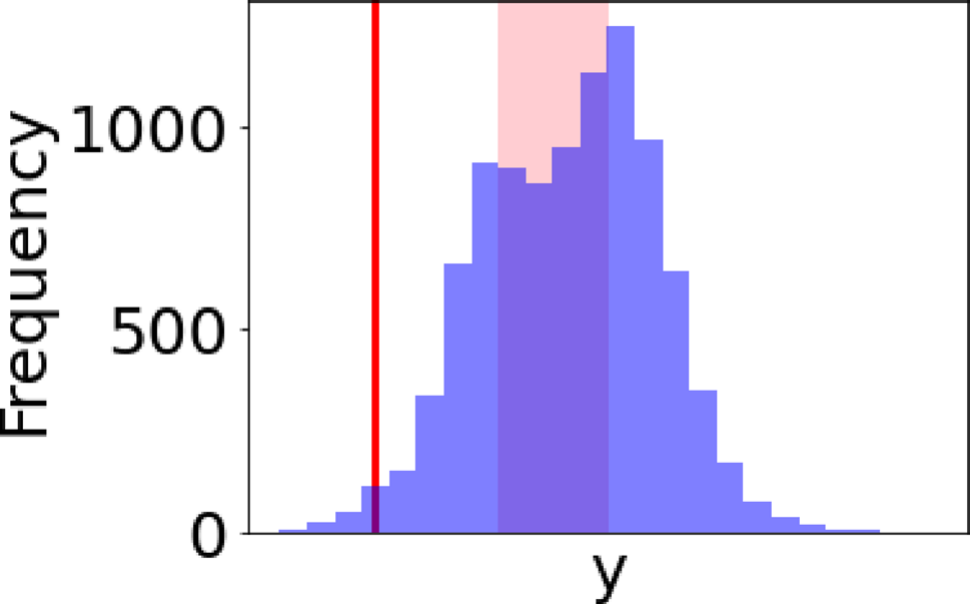
Histogram of the predicted Y values of the virtual samples after the inverse analysis in the pitch coke production process. The light red area is the target area, and the red line is the measured value of Y for the raw material sample of the target

To perform inverse analysis of the constructed models, with the variables related to the raw materials fixed at the values of the sample of historical data with the smallest value of Y, 10,000 samples of time series data with controllable process conditions were generated using T-Gen. The generated samples were input to the constructed model, and Y values were predicted. There existed 9614 samples in the AD set with the 5-nearest neighbor algorithm. A histogram of the predicted Y values for the 9614 samples is shown in Fig. 7. The area in light red represents the target area of Y, and the red line represents the actual measured value of Y for the samples with fixed raw materials. There existed 3837 samples in which Y was within specification using raw materials of products whose Y values were out of specification in the past. We could design the time series data of process conditions to control Y within the specification by using the proposed method.

**Fig. 8:**
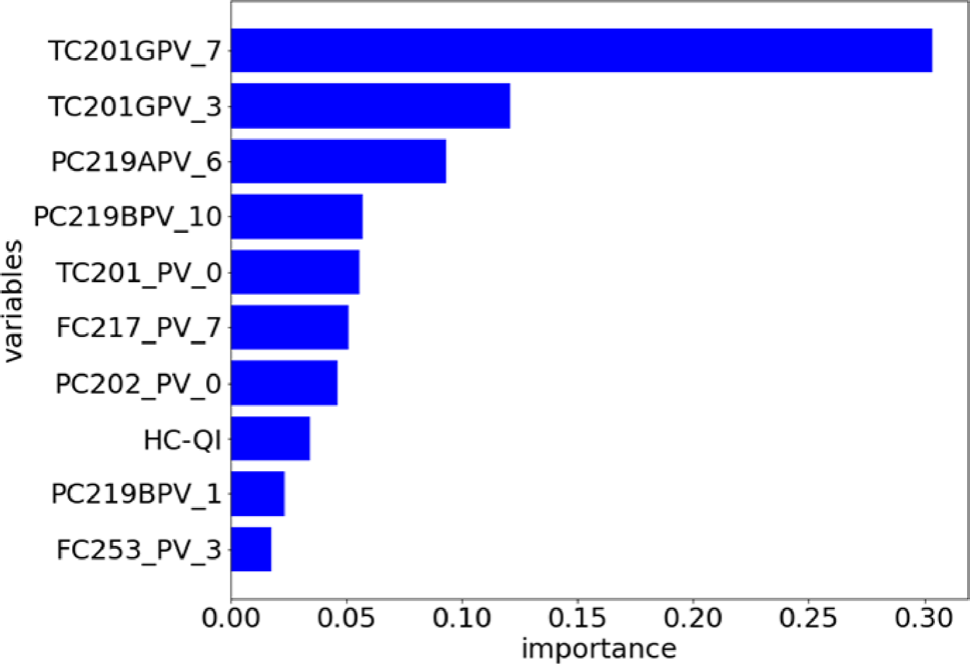
Results of the variable importance of the model using CVPFI in the pitch coke production process

**Fig. 9:**
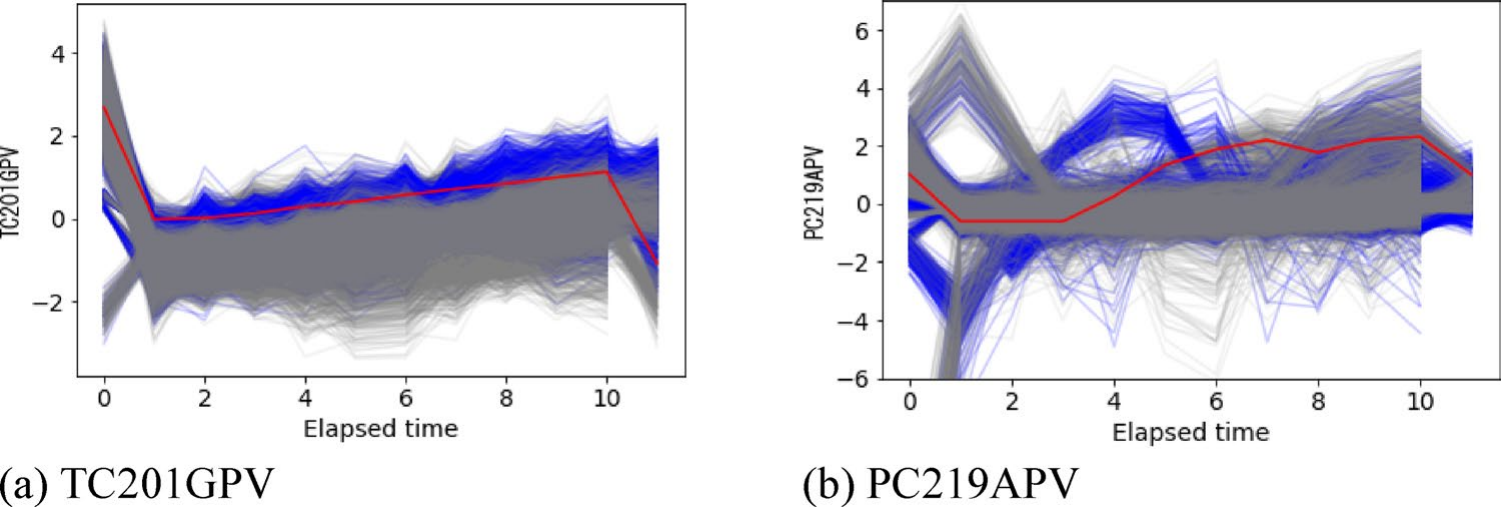
Time series data of the top two process conditions in terms of variable importance in the pitch coke production process. Red, blue and gray lines mean historical time series using the raw material used for the inverse analysis, time series estimated to be within specifications, and time series estimated to be outside specifications, respectively. (**a**) TC201GPV, (**b**) PC219APV

Figure 8 shows the calculation results of the variable importance of the model using CVPFI for the top 10 variables with the high importance. The names of X are expressed as a combination of the name of the manufacturing condition and the point in time from the start of the batch time (“manufacturing condition"_"elapsed time from batch start point”). Figure 9 shows the results of visualizing the time series data of the top two process conditions from the variables considered important by CVPFI. For the most important variable, elapsed time 3 and 7 in Fig. 8 (a), product properties tend to be outside the target range when the value of the variable is low. Figure 9(b) shows a characteristic change over time for variables with high values of CVPFI. It is estimated that data with a time series of constant values or data with a behavior of increasing values at batch elapsed time of 6 or 8 cannot keep the product properties within the target range. This result was similar to the past samples with fixed raw material properties indicated by the red line. On the other hand, when the batch elapsed time 3 and 4 was increased, it was confirmed that the product properties are within the target range.

## Conclusions

This study developed machine learning models to predict product properties from raw material properties and process conditions using actual operating data of needle and pitch coke manufacturing processes. The proposed method, NSGA-II-VDS, combined GAVDS, which selects important process conditions and their time delays for the prediction of objective variables, and NSGA-II, which is effective for the optimization of two or more fitness functions in the needle coke manufacturing process. For two objective variables, the proposed NSGA-II-VDS showed higher prediction accuracy in optimization and prediction of test data than the conventional GAVDS. For the pitch coke production process, product property prediction model was developed using a data set with aligned data lengths to account for the different batch time length characteristics of samples produced in a batch process. The highest prediction accuracy was achieved with a data set in which values at unmanufactured times for short batches were interpolated by the average value for that batch. Furthermore, by generating virtual time-series data and inputting them into the prediction model developed, it was possible to propose process conditions that would yield the desired product property values, based on the predicted Y values. Then, we could find characteristic behaviors of the time-series data that yielded the desired product property values from changes over time in the process conditions of a large number of virtual samples. It is expected that the proposed methods can be used to improve the efficiency of coke production processes.

## Data Availability

The data that support the findings of this study are not publicly available due to confidentiality restrictions under a non-disclosure agreement with an industrial partner.
